# Teaching Gender Differences at Medical School Could Improve the Safety and Efficacy of Personalized Physical Activity Prescription

**DOI:** 10.3389/fcvm.2022.919257

**Published:** 2022-06-23

**Authors:** Anna Vittoria Mattioli, Milena Nasi, Marcello Pinti, Carla Palumbo

**Affiliations:** ^1^Department of Surgery, Medicine, Dentistry and Morphological Sciences, University of Modena and Reggio Emilia, Modena, Italy; ^2^Department of Life Sciences, University of Modena and Reggio Emilia, Modena, Italy; ^3^Department of Biomedical, Metabolic, and Neural Sciences, University of Modena and Reggio Emilia, Modena, Italy

**Keywords:** gender medicine, sports, teaching, gender bias, cardiovascular disease

## Introduction

The most recent scientific evidence on gender medicine indicates that the teaching of gender medicine is fundamental in the training of doctors and health professionals. This opinion paper aims to focus attention on the benefits of systematic teaching of gender medicine to improve the safety and efficacy of prescribing personalized physical activity and sport.

The practice of physical activity and sport has become extremely widespread in the elderly adult population also following the indications of the WHO Global Action Plan on Physical Activity 2018–2030 which indicates the prescription of physical activity for the prevention of non-communicable diseases (1).

A recent manuscript points out that implicit prejudice education is effective in raising awareness of prejudice regardless of personal beliefs (2).

Raising awareness of bias is one of many steps toward creating a positive learning environment and a more equitable healthcare system (2).

## Gender Medicine

Gender bias in medicine persists and affects patient care, trainee assessment, and organizational climate (3).

Cardiovascular (CV) diseases are an important example because they have different characteristics in both symptoms and prognosis in women and men (4).

The study of gender medicine in the CV field has led to the identification of specific CV risk factors for women related to hormonal life and hormonal changes over time.

Risk factor analysis is an example of how gender medicine can modify CV risk stratification. Traditional risk factors have been studied on predominantly male populations inducing the erroneous belief that the only relevant risk factor in women is menopause. Indeed, menopause acts as an important risk factor because it is associated with a series of modifications of traditional risk factors such as obesity. With menopause, there is a change in obesity that becomes visceral obesity and the appearance of the phenomenon known as sarcopenic obesity (5).

Other specific risk factors for women have been identified: i.e., hypertension and gestational diabetes. In addition to this, the same traditional risk factors are known to carry a different weight in women than in men. An important role is also the social and economic conditions.

Throughout a woman’s life, hormonal variations affect exposure to different sex-specific risk factors at different ages. The best-known example is menopause, which marks an increase in the CV risk in women caused by the modification of the hormonal and metabolic balance (4). The menopausal woman develops visceral obesity, an important CV risk factor. During the fertile period, pregnancy is a critical moment. In recent years it has emerged that the onset of diseases, such as gestational diabetes and hypertension, affects a future risk of developing atherosclerosis (4, 6).

An independent risk factor for maternal and neonatal morbidity and mortality is pre-pregnancy obesity (7). Maternal obesity is associated with a higher risk of gestational diabetes, hypertension, preeclampsia, and adverse birth outcomes with an increased risk of cesarean delivery and wound complications (7).

Physical activity is an excellent tool to fight against obesity, and women must use it both during pregnancy and after menopause. The World Health Organization (WHO) recommends that “women who, before pregnancy, habitually engaged in a vigorous-intensity aerobic activity or who were physically active, can continue these activities during pregnancy and the postpartum period” (8). Additionally, encouraging pregnant women to engage in exercise programs is crucial in managing their weight gain and maintaining a healthy lifestyle (9).

Women are less likely to engage in physical activity and sports from an early age affected by an ancient social role (10). A sedentary lifestyle is more common in women and appears to have a greater CV effect in women than men. The recent pandemic has shown that women have suffered from increased stress, which has been reflected in poor diet, reduced physical activity, and increased sedentary lifestyle (11, 12). Psychosocial disadvantages (e.g., unemployment, chronic stress, insufficient social support, and social isolation) are more common in women than in men, which contributes to increased depression and anxiety (13). Moreover, the situation worsened significantly during the pandemic (13, 14).

Socio-economic factors affect CV risk with different effects in women and men. The knowledge of these differences would determine an adequate stratification of the risk to introduce tailored prevention actions (13, 14).

Many factors contribute to the gender bias in diagnosis and management of CVD: gender disparity in clinical evidence studies, which have a low percentage of women, the lack of CV disease research specifically dedicated to women, insufficient education specifically aimed at women on CV risk factors specific to women, underestimation by the national health system the prevalence of CVD among women, under evaluation of symptoms, and poor perception of subjective risk (15, 16) (Table 1).

**TABLE 1:** Gender differences in the cardiovascular field that could be corrected by teaching gender medicine.

1. Cardiovascular diseases are underdiagnosed and undertreated in women.
2. Women experience an inappropriate reduction in the dosage of cardiovascular drugs, especially if they are elderly.
3. Women are less likely to be screened for cardiovascular risk factors for prevention.
4. The pathophysiological mechanisms for cardiovascular disease in women and sex-specific cardiovascular risk factors are poorly studied.
5. The impact of psychosocial and socioeconomic factors on cardiovascular risk burden in women is underestimated.
6. There is a reduced perception of cardiovascular risk in women.

The lack of gender-specific clinical guidelines can adversely affect patient care, particularly for women with hypertension or diabetes, for whom therapy often fails to meet its goals (17).

A recent position paper underlines that sex and gender should be incorporated into the design of prospective trials to ensure that outcomes and the implementation of findings are broadly and appropriately applicable to patient care (17).

In recent years, medical awareness of CV risk in women has increased, somewhat reducing the phenomenon of underdiagnosis of CVD. However, most healthcare professionals and female patients themselves still tend to underestimate the CV risk in women (4).

Although the same proportion of women and men present with chest pain of CV origin in ambulatory care, there is a strong sex bias in their management; men were 2.5 times more likely to be referred to a cardiologist than women. These data suggest that effort must be made to assure equality between men and women in medical care (18).

Moreover, several studies have shown that the perception of CV risk in women themselves has not changed, despite intense information and prevention campaigns (19). Sex-specific mechanisms in the pathophysiology of CV disease in women remain poorly understood.

Gender-dependent differences in morphological and functional aspects of body composition, as well as the well-recognized sexual dimorphisms in metabolic substrates, not only influence the risk, diagnosis, and therapeutic management of CVD, but also cardiac rehabilitation, e.g., after coronary artery bypass grafting (CABG) (20, 21).

Gender, besides body composition and age, strongly affects cardiac rehabilitation; older women post-cardiac surgery are characterized by a higher disability index in relation to tolerance to physical stress in comparison with men of the same age (21).

All these subjective, as well as objective, factors contribute to invalidating efforts in the field of prevention and in encouraging the adoption of healthy lifestyles and therapies (22).

How to counter these established and unhealthy habits? An effective strategy must include a different approach in women than in men and the basis for this strategy must include the systematic teaching of gender medicine.

## Gender Bias in Medical Education

In a review of gender bias in resident physician assessment, five out of nine studies found that gender bias potentially influenced the assessment of resident physicians (23).

Medical schools now average approximately 50% female students, yet a disproportionate number of women continue to choose non-surgical over surgical specialties (23, 24). Once in training, studies indicate that pervasive gender stereotypes, sexism, and harassment negatively affect female surgeons (24).

Gender bias leads to negative experiences for women trainees in a surgical specialty, and women trainees in the male-dominated specialty are more prone to leave medicine and retire early (18). Previous studies have offered theories as to why women do not enter surgical specialties or assume leadership roles (23, 24). These include a paucity of role and family models’ responsibilities (25). However, these studies did not take into account the influence of gender discrimination in discouraging women from pursuing surgery or surgical sub-specialty (25).

Furthermore, men continue to outnumber women in most medical specialties, and in some of the larger specialties, there are three times as many men as women. As an example: in cardiology, 82% of doctors are men, as well as in gastroenterology and hepatology where 73% of doctors are men (26). Overall, 58% of consultants and higher specialization trainees (aged 3–8) are men, although in some major specialties, there is a more uniform gender division (26).

## Gender Medicine in Sports and Physical Activity

Specific information on the different structures of muscle and joints, the different responses of the muscle to physical activity, and the different musculoskeletal pathology in women and men help to create a culture of personalized non-pharmacological prescriptions for physical activity.

The effectiveness of the systematic teaching of a procedure has been extensively studied in cardiopulmonary resuscitation where the spread of teaching resuscitation maneuvers has resulted in an improvement in response and survival in out-of-hospital cardiac arrest (27, 28).

Several studies showed that men and women adapt differently to endurance exercise (29, 30). Nonetheless, relative to body size, men and women can achieve comparable gains in strength and fitness after exercise training (27, 30). However, the mechanisms driving sex-specific adaptation to exercise are largely unknown.

It is also well-known that men and women differ in substrate utilization during endurance exercise. Many studies have shown that women rely less on carbohydrate and protein sources and more on fat sources to support substrate oxidation during endurance exercise (31). These sex differences may partly be explained by estrogen concentration and activity (32).

Moreover, there are sex differences in fiber type distribution and cross-sectional area in many muscle groups (33).

Women tend to have a higher percentage of slower type I and IIA fibers compared with men. This reflects the lower contractile velocity and the enhanced fatigue resistance of women, as oxidative fibers allow for enhanced endurance and recovery. Despite the multitude of research conducted on exercise genomics, there is still a lack of knowledge on specific genes. The reason is that exercise-related phenotypes are polygenic and influenced by many other factors, which make it challenging to identify genes with large effect sizes (33). Sex-based differences in exercise muscle metabolism strongly impact exercise and nutritional strategies: all these parameters/conditions are to be taken into account to optimize not only performance in women but also, and more importantly, the overall women’s health (20).

Gender medicine is not yet widespread in medical education; however, it is essential to move in this direction (34).

The increase in knowledge will lead to a more effective approach. Moreover, one of the risks of spontaneous physical activity is CV safety. The stratification of the risk level of developing events must also be based on the different weight of traditional CV risk factors, on the assessment of emerging risk factors, and on the different pathophysiological responses of the musculoskeletal system in the two sexes (35). Similarly, inflammatory and immunologic responses are different in the two sexes.

It is also well-known that there are differences in the development of arrhythmias and the epidemiology of channelopathy. Cellular electrophysiological sex differences have been found for the action potential sodium, calcium, and potassium currents, which affect both depolarization and repolarization cycles (9).

Women have a higher risk of drug-related arrhythmia from drugs that block potassium currents in the cardiomyocyte channel. These drugs include not only antiarrhythmic drugs but also CV and non- CV drugs, such as antidepressants, antifungals, and antihistamines (9).

It is essential that sports and health professionals know these differences to identify early the subjects at risk; assuming that the different risk between men and women is determined not only by the influence of hormones but also by the environment and genetics (36).

The recent pandemic has led to important developments in teaching and distance learning, as well as in university and post-graduate training (37, 38).

With the rise of innovative virtual learning solutions, medical educators will need to leverage technology to develop e-learning materials that facilitate effective adult learning. This approach should include all issues relevant to medical education including gender medicine. The use of technology-based training solutions has been approved in several guidance documents for CV medicine trainees in several countries (39). Recommendations have been published to better address the challenges of digital health implementation in CV medicine (40). These experiences, gained during such a dramatic moment for public health, must be adapted to the post-pandemic reality in order not to lose valuable tools for learning.

A new frontier will be health and safety for transgender athletes. The gender-affirming medical and surgical treatments and the unique ways the musculoskeletal system could be affected by each, such as impaired bone health, changes in ligament function, and the potential increased risk of deep venous thromboembolism, are essential for providing optimal musculoskeletal care for transgender athletes (41, 42).

## Conclusion

We must act promptly with a series of actions on medical school programs adapted to sex differences in medical practice in order to reduce the gap between men and women. Physical activity therapy prescription should be personalized and take into account gender differences in muscle and joint structure to optimize the outcome.

## Author Contributions

AM, MN, MP, and CP contributed to the conception, design of the work, draft, and the final approval of the manuscript. All authors contributed to manuscript revision, read, and approved the submitted version.

## Conflict of Interest

The authors declare that the research was conducted in the absence of any commercial or financial relationships that could be construed as a potential conflict of interest.

## Publisher’s Note

All claims expressed in this article are solely those of the authors and do not necessarily represent those of their affiliated organizations, or those of the publisher, the editors and the reviewers. Any product that may be evaluated in this article, or claim that may be made by its manufacturer, is not guaranteed or endorsed by the publisher.
